# Onset of brain injury in infants with prenatally diagnosed congenital heart disease

**DOI:** 10.1371/journal.pone.0230414

**Published:** 2020-03-25

**Authors:** Mirthe J. Mebius, Catherina M. Bilardo, Martin C. J. Kneyber, Marco Modestini, Tjark Ebels, Rolf M. F. Berger, Arend F. Bos, Elisabeth M. W. Kooi

**Affiliations:** 1 Division of Neonatology, Beatrix Children’s Hospital, University of Groningen, University Medical Center Groningen, Groningen, The Netherlands; 2 Department of Obstetrics & Gynecology, University of Groningen, University Medical Center Groningen, Groningen, The Netherlands; 3 Division of Pediatric Critical Care Medicine, Beatrix Children’s Hospital, University of Groningen, University Medical Center Groningen, Groningen, The Netherlands; 4 Critical Care, Anesthesiology, Peri-operative & Emergency medicine (CAPE), University of Groningen, Groningen, The Netherlands; 5 Department of Anesthesiology, University of Groningen, University Medical Center Groningen, Groningen, The Netherlands; 6 Center for Congenital Heart Diseases, Department of Cardiothoracic Surgery, University of Groningen, University Medical Center, Groningen, The Netherlands; 7 Center for Congenital Heart Diseases, Pediatric Cardiology, Beatrix Children’s Hospital, University of Groningen, University Medical Center Groningen, Groningen, The Netherlands; Western University, CANADA

## Abstract

**Background:**

The exact onset of brain injury in infants with congenital heart disease (CHD) is unknown. Our aim was, therefore, to assess the association between prenatal Doppler flow patterns, postnatal cerebral oxygenation and short-term neurological outcome.

**Methods:**

Prenatally, we measured pulsatility indices of the middle cerebral (MCA-PI) and umbilical artery (UA-PI) and calculated cerebroplacental ratio (CPR). After birth, cerebral oxygen saturation (r_c_SO_2_) and fractional tissue oxygen extraction (FTOE) were assessed during the first 3 days after birth, and during and for 24 hours after every surgical procedure within the first 3 months after birth. Neurological outcome was determined preoperatively and at 3 months of age by assessing general movements and calculating the Motor Optimality Score (MOS).

**Results:**

Thirty-six infants were included. MOS at 3 months was associated with MCA-PI (rho 0.41, *P* = 0.04), UA-PI (rho -0.39, *P* = 0.047, and CPR (rho 0.50, *P* = 0.01). Infants with abnormal MOS had lower MCA-PI (*P* = 0.02) and CPR (*P* = 0.01) and higher UA-PI at the last measurement (*P* = 0.03) before birth. In infants with abnormal MOS, r_c_SO_2_ tended to be lower during the first 3 days after birth, and FTOE was significantly higher on the second day after birth (*P* = 0.04). Intraoperative and postoperative r_c_SO_2_ and FTOE were not associated with short-term neurological outcome.

**Conclusion:**

In infants with prenatally diagnosed CHD, the prenatal period may play an important role in developmental outcome. Additional research is needed to clarify the relationship between preoperative, intra-operative and postoperative cerebral oxygenation and developmental outcome in infants with prenatally diagnosed CHD.

## Introduction

Up to 50% of infants with congenital heart disease (CHD) have neurodevelopmental impairments later in life [1]. As a consequence, many adults with CHD experience psychosocial and cognitive challenges that could affect quality of life [2,3]. Increasing evidence suggests that neurodevelopmental impairments in infants with CHD may result from insults occurring from as early as the second trimester.

Prenatally, circulatory alterations and impaired brain maturation have been frequently observed [4]. Fetuses with CHD have increased cerebral blood flow [5,6], decreased cerebral vascular resistance [5–9], smaller head circumference [7,8,10], lower total brain weight [11], impairments in sulcation [12,13], altered cerebral metabolism [13,14], and abnormalities on MRI that are in accordance with impaired brain maturation such as ventriculomegaly and increased extra-axial cerebrospinal fluid spaces [15,16].

After birth, many of the prenatal findings, such as a smaller head circumference, lower total brain volumes, and an altered cerebral metabolism persist [4,17–21]. Furthermore, in comparison with healthy newborns, infants with CHD have lower cerebral oxygen saturation [22,23], more neurobehavioral abnormalities [24], increased epileptic activity [25,26] and up to 53% show brain abnormalities on MRI prior to surgery [27–29]. The most commonly observed abnormalities include white matter injury, and stroke [27–29].

Surgical procedures and the postoperative period pose an additional threat to the young brain. Ischemia and reperfusion injury, hypothermia, inflammatory and immune responses, altered cerebral blood flow regulation and decreased cardiac output might all contribute to brain injury during and after surgery [30,31]. New brain abnormalities on MRI after cardiac surgery are reported in up to 78% of infants with CHD [32,33].

Currently, it is still unknown which period is the most threatening for the young developing brain in infants with CHD. To date, no study has assessed cerebral abnormalities and its relation with neurological outcome from before birth to the postoperative period in these infants. Our aim was, therefore, to perform a longitudinal assessment of prenatal Doppler flow patterns and postnatal, intraoperative and postoperative cerebral oxygen saturation and extraction in relation to short-term neurological outcome in infants with CHD that were admitted to the intensive care (ICU) immediately after birth.

## Methods

### Study population

A prospective observational cohort study (registration number: NTR5523) was conducted at the fetal medicine unit, the NICU, congenital heart center and pediatric intensive care unit of the University Medical Center Groningen. Between May 2014 and August 2016, all fetuses with isolated CHD expected to require ICU admission immediately after birth were prenatally enrolled when parental informed consent was obtained. After birth, infants were echocardiographically assessed by a pediatric cardiologist to confirm the cardiac diagnosis. Infants were excluded from further participation if cardiac diagnosis could not be confirmed, if born before a gestational age of 36 weeks, or in case of major chromosomal, genetic or structural anomalies that became apparent after birth. This study was approved by the Medical Ethical Committee of the University Medical Center Groningen METc number: METc2014/083.

### Neurological outcome

Neurological outcome was assessed by general movements (GMs) at two different moments. Preoperatively, at the age of 7 days (5–10 days), a video recording of 30–60 minutes was made. Postoperatively, at the age of 3 months, a video recording of approximately 10 minutes was made [34]. During the recordings, the infants wore a diaper or body to ensure visibility of movements. Video recordings during crying or sucking on a dummy were excluded from the analysis.

The GMs were scored independently by two different assessors (MJM and AFB) and one of them was unaware of the clinical condition of the patient (AFB). Both observers are certified as advanced scorers by the GM Trust. At the age of 7 days, the GMs were scored as either normal or abnormal. Abnormal GMs included poor repertoire, chaotic, and cramped synchronized movement patterns. Furthermore, we explored more detailed aspects of the motor repertoire reflected in a motor optimality score (MOS). At this age, the MOS ranges from 8 (poor) to 18 (optimal) and MOS <15 was considered to be abnormal. At the age of 3 months we assessed the presence and quality of fidgety movements (normal/abnormal/absent). Furthermore, we assessed the MOS which ranges from 5 (poor) to 28 (optimal) at this age and MOS <25 was considered to be abnormal [35–36].

### Doppler flow patterns

During pregnancy, pulsatility indices of the middle cerebral artery (MCA-PI) and umbilical artery (UA-PI) were measured repeatedly by an experienced fetal medicine expert (CMB) and cerebroplacental ratio (CPR) was calculated. All Doppler parameters were converted into Z-scores to adjust for differences in gestational age at fetal examinations. For statistical purposes, the last available Doppler measurement before birth was used.

### Near-infrared spectroscopy

After birth, cerebral oxygen saturation (r_c_SO_2_) was measured with the INVOS 5100c spectrometer (Medtronic, Dublin, Ireland) with neonatal sensors (Medtronic), placed on the frontoparietal side of the forehead. First, r_c_SO_2_ was measured daily for at least two consecutive and stable hours during the first 3 days after birth starting immediately after stabilization of the infant on the NICU. Preferably, the measurements were performed at the same time each day to avoid possible effects of diurnal variation. Second, r_c_SO_2_ was measured during every corrective/palliative surgical procedure within the first 3 months after birth. Third, r_c_SO_2_ was measured for 24 hours following each invasive cardiac procedure within the first 3 months after birth. Simultaneously with r_c_SO_2_ measurements, we measured preductal arterial oxygen saturation (SpO_2_) and calculated cerebral fractional tissue oxygen extraction (FTOE). For statistical purposes, we selected representative two-hour periods of stable r_c_SO_2_ measurements, preferably at the same time during the day, and calculated mean r_c_SO_2_ and FTOE for each of the first 3 days after birth. In addition, we calculated mean r_c_SO_2_ and FTOE during cardiac surgery and for 24 hours after cardiac surgery. Furthermore, we assessed the burden of hypoxia in two ways (percent of time <60% and percent of time <50%) and r_c_SO_2_ nadir during cardiac surgery.

### Statistical analysis

For statistical analyses, we used SPSS version 23.0 (IBM Corp., Armonk, NY, USA) and for graphical display GraphPad Prism version 5 was used. Data are presented as either median (range) or number (percentage). First, we used descriptive statistics to visualize the association between fetal Doppler flow patterns, postnatal, intraoperative and postoperative r_c_SO_2_ and FTOE and short-term neurological outcome. Second, we used Spearman’s correlation coefficient or Mann-Whitney U test to assess the association between short-term neurological outcome and fetal Doppler flow patterns, r_c_SO_2_ and FTOE. Third, we categorized fetal Doppler flow patterns, postnatal r_c_SO_2_, intraoperative r_c_SO_2_ and postoperative r_c_SO_2_ as being either normal or abnormal and used Fisher’s exact test to assess the association between the number of abnormal values and short-term neurological outcome. Abnormal prenatal Doppler flow patterns were defined as Z-scores >1.0 or <-1.0. Abnormal r_c_SO_2_ was defined as values <60% or >90% [37–38]. The lower r_c_SO_2_ cut-off value was based on the hypoxia-ischemia threshold of 33–44% for functional impairment. As these values were measured with a sensor that measures approximately 10% lower than the sensors we used, we chose for 60% as the lower cut-off value [37]. The higher cut-off value was based on Verhagen et al. who found that values >90% were associated with poorer outcome [38]. A *P*-value <0.05 was considered significant.

## Results

### Patient characteristics

**Fig 1:**
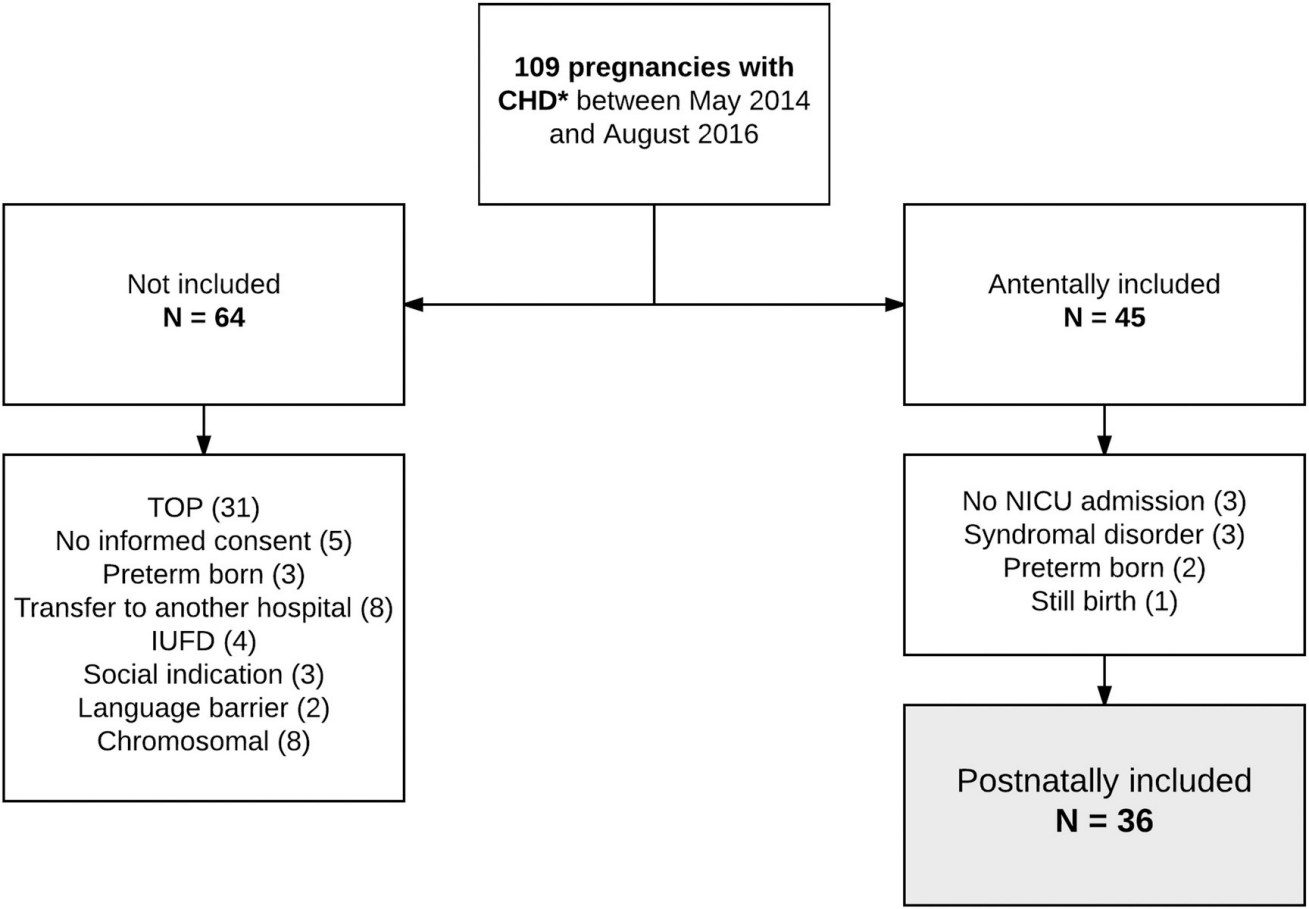
Flow chart inclusion and exclusion. CHD, congenital heart disease; TOP, termination of pregnancy; IUFD, intrauterine fetal demise; NICU, neonatal intensive care unit. * Cardiac lesions that require birth at a congenital heart center.

Initially, 45 fetuses with CHD were included between May 2014 and August 2016 (Fig 1). After birth, nine neonates were excluded, as they did not meet the inclusion criteria. Three infants were not admitted to the ICU, three infants were excluded due to chromosomal or genetic abnormalities (45XO, duplication chromosome 7 and Kabuki syndrome), two infants were excluded because of preterm birth and one fetus was stillborn. Gestational age at birth was 39.1 (36.6–40.3) weeks and birth weight was 3535 (2100–4120) grams. One infant had an Apgar score <6 at 5 minutes with a pH of the umbilical artery of 7.23. Shortly after birth, one infant presented with a brief period of persistent pulmonary hypertension of the neonate. Seven infants died; one because of massive myocardial infarction prior to surgery, two because of inoperable cardiac lesions, two because of severe brain injury after cardiopulmonary resuscitation, and two infants due to other (non-cardiac) reasons. Twenty-six infants (72%) underwent cardiac surgery during the study period. Median (range) number of interventions was 1 (1–3) and three infants (8%) between 2 and 27 days after birth. Patient characteristics are presented in Table 1 and detailed aspects of the included cardiac lesions are presented in S1 Table.

**Table 1:** Patient characteristics.

	*N = 36*
Gestational age at birth (weeks)	39.1 (36.6–40.3)
Birth weight (grams)	3535 (2100–4120)
Birth weight <10^th^ percentile	6 (16)
Male	21 (58)
**Type of CHD**	
• TGA • HLHS • Pulmonary stenosis • Pulmonary atresia • Coarctation of the aorta • Tetralogy of Fallot • Common arterial trunk • Complex CHD • AVSD • Tricuspid dysplasia • DORV	9 (25) 4 (11) 1 (3) 2 (6) 4 (11) 4 (11) 3 (8) 4 (11) 1 (3) 1 (3) 3 (8)
Apgar at 5 minutes	8 (5–10)
Mortality	7 (19)
MABP day 1	44 (37–51)
MABP day 2	47 (34–56)
MABP day 3	46 (42–54)
**Respiratory support day 1** (n = 35)	
• None/low flow • CPAP • NIPPV • SIMV/SIPPV	19 (54) 9 (26) 1 (3) 6 (17)
**Respiratory support day 2** (n = 34)	
• None/low flow • CPAP • NIPPV • SIMV/SIPPV	19 (56) 3 (9) 2 (6) 10 (29)
**Respiratory support day 3** (n = 32)	
• None/low flow • CPAP • NIPPV • SIMV/SIPPV	20 (63) 3 (9) 2 (6) 7 (22)
**Medical treatment**	
Prostaglandin E_1_ day 1 (n = 35)	22 (63)
Prostaglandin E_1_ day 2 (n = 34)	22 (65)
Prostaglandin E_1_ day 3 (n = 32)	21 (66)
Sedatives day 1 (n = 35)	8 (23)
Sedatives day 2 (n = 34)	15 (44)
Sedatives day 3 (n = 32)	12 (38)
Placental insufficiency (pathology report)	4 (11)
Abnormality cerebral echocardiogram	7 (19)
ICU stay (days)	10 (4–90)
Age at surgery (days)	9 (2–27)

Data represent either median (range) or number (percentage). CHD, congenital heart disease; TGA, transposition of the great arteries; HLHS, hypoplastic left heart syndrome; AVSD, atrioventricular septal defect; DORV, double outlet right ventricle; MABP, mean arterial blood pressure; CPAP, continuous positive airway pressure; NIPPV, nasal intermittent positive pressure ventilation; SIMV, synchronized intermittent mandatory ventilation; SIPPV, synchronized intermittent positive pressure ventilation; ICU, intensive care unit.

### Neurological outcome

Preoperatively, GMs were recorded in 25 infants. Fourteen infants (56%) had abnormal GMs. They all scored poor repertoire, none had chaotic or cramped synchronized movements. Motor optimality score was 13 (10–18). In eleven infants, GMs were not recorded due to scheduled surgery prior to the age of five days (n = 4), immobility due to sedation (n = 1), transfer to another hospital within five days after birth (n = 3) and mortality (n = 3).

At the age of three months, GMs were recorded in 29 infants. Two infants had absent fidgety movements and one had abnormal fidgety movements. Motor optimality score was 26 (11–28). Based on the cut-off point of <25, twelve infants (41%) had abnormal motor optimality scores. Reasons for missing recordings at this age were mortality before the age of three months (n = 6) and withdrawal from study participation (n = 1).

Twenty-one infants had GMs assessment at both ages. Of these infants, ten remained stable, three deteriorated and eight improved (Table 2). McNemar test confirmed that the trend of deterioration of the infants over time was not significant (*P =* 0.25). Two of the three infants with deteriorating neurological outcome had a complicated postoperative course. One infant had low cardiac output syndrome and the other infant developed a cardiac tamponade needing a re-intervention.

**Table 2:** The course of general movements in infants with CHD.

Infant	GM 7	GM 3	Change	Infant	GM 7	GM 3	Change
**1**†	x	x	na	**19**†	A	x	na
**2**	x	N	na	**20**	N	N	=
**3**	A	N	↑	**21**	A	A	=
**4**†	N	x	na	**22**	x	A	na
**5**	A	N	↑	**23**	A	N	↑
**6**	A	N	↑	**24**	N	N	=
**7**	x	A	na	**25**†	A	x	na
**8**†	N	x	na	**26**†	x	x	na
**9**	N	A	↓	**27**	A	A	=
**10**	N	N	=	**28**	x	A	na
**11**	A	N	↑	**29**	A	N	↑
**12**	N	N	=	**30**	x	x	na
**13**	x	A	na	**31**	A	N	↑
**14**	x	N	na	**32**	N	N	=
**15**	x	N	na	**33**	N	A	↓
**16**	N	N	=	**34**	N	A	↓
**17**	A	A	=	**35**	A	A	=
**18**	x	A	na	**36**	A	N	↑

GM 7, preoperative general movements at the age of 7 days; GM 3, postoperative general movements at the age of 3 months; x, not recorded; N, normal assessment; A, abnormal assessment; na, not applicable; †, infant died before the age of 3 months.

### Fetal cerebral vascular resistance and neonatal cerebral oxygen saturation

Prenatal Doppler flow patterns were assessed in all infants. Gestational age at the last fetal examination was 34.4 (21.1–39.1) weeks. In comparison with reference values of healthy fetuses, we found slightly lower MCA-PI (-0.19 (-3.94 to 2.59), *P* = 0.35) and CPR (-0.65 (-4.06 to 2.80), *P* = 0.01) and slightly higher UA-PI Z-scores (0.44 (-1.74 to 4.01), *P* = 0.02).

After birth, r_c_SO_2_ increased from 61% (32%-89%) to 70% (52%-91%) during the first 3 days after birth. On day 1, six neonates had r_c_SO_2_ values <50%, which could be explained by clinical conditions (restrictive foramen ovale, fetal-maternal transfusion or perinatal asphyxia). All neonates stabilized during the first 3 days and none of the neonates had r_c_SO_2_ values <50% on day 3 after birth. Twenty-six neonates (72%) had at least one cardiac surgical procedure within the first 3 months after birth. During surgical procedures, r_c_SO_2_ was lower in comparison with the first 3 days after birth (53% (36%-69%)). Median duration of surgery was 7.0 (1.5–12.3) hours. The median burden of hypoxia <60% and <50% was 68% (4%-100%) and 34% (0%-95%) of the recording time, respectively. The median r_c_SO_2_ nadir during surgery was 22% (15%-56%). Following cardiac surgery, r_c_SO_2_ increased to 61% (42%-78%).

### Timing of occurrence of brain injury

Prenatal Doppler flow patterns strongly correlated with MOS at 3 months of age. Pulsatility index of the middle cerebral artery (rho 0.41, *P* = 0.04) and CPR (rho 0.50, *P* = 0.01) positively correlated with MOS, while UA-PI negatively correlated (rho -0.39, *P* = 0.04) with MOS at 3 months of age. There were no significant correlations between postnatal, intraoperative (mean, burden of hypoxia and nadir) and postoperative r_c_SO_2_ or FTOE and MOS at the age of 3 months.

**Fig 2:**
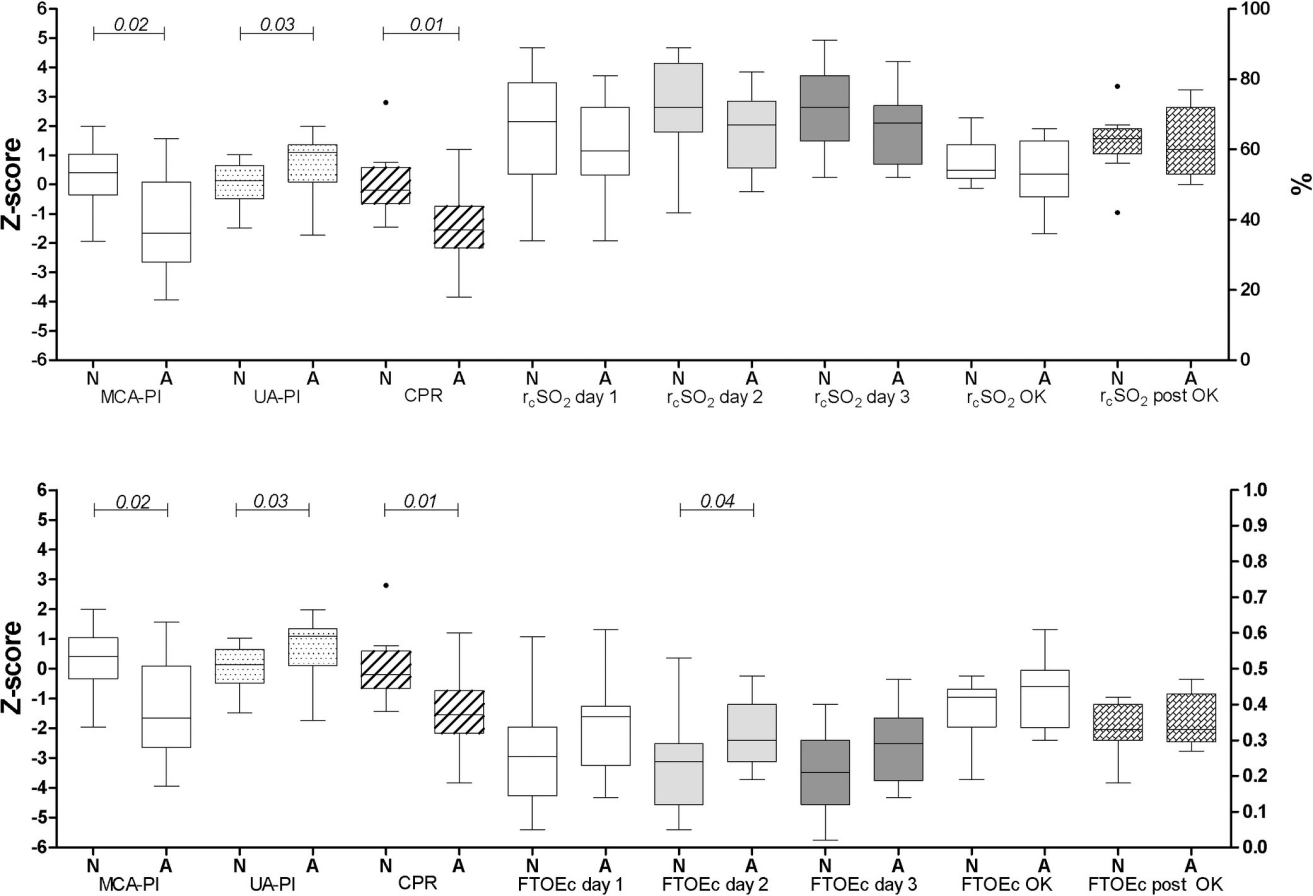
Fetal Doppler flow patterns, postnatal r_c_SO_2_ and FTOE according to GMs assessment at the age of 3 months. Data are shown in box-and-whisker plots. Circles represent outliers. N, normal general movements based on MOS ≥25; A, abnormal general movements based on MOS <25; MCA-PI, pulsatility index of the middle cerebral artery; UA-PI, pulsatility index of the umbilical artery; CPR, cerebroplacental ratio; r_c_SO_2_, regional cerebral oxygen saturation; FTOE, fractional tissue oxygen extraction.

Results were similar for abnormal and normal MOS (Fig 2 and S2 Table). Infants with abnormal MOS had lower MCA-PI (*P* = 0.02), higher UA-PI (*P* = 0.03) and lower CPR (*P* = 0.01) in comparison with infants with normal MOS at the age of 3 months. Cerebral oxygen saturation during the first 3 days after birth was lower in infants with abnormal MOS, however, this did not reach statistical significance. Both during and after surgical procedures r_c_SO_2_ (mean, burden of hypoxia and nadir) was similar in infants with normal and infants with abnormal MOS. In infants with abnormal MOS, FTOE tended to be higher during the first three days after birth, reaching statistical significance on day 2 (*P* = 0.04). During and after cardiac surgical procedures, there were no associations between FTOE and short-term neurological outcome. The number of interventions was also not associated with GMs at an age of 3months (*P* = 0.12).

Infants with a combination of abnormal prenatal and postnatal cerebral values were more likely to have abnormal MOS in comparison with infants that had no abnormal cerebral values or only at one of both periods (odds ratio = 9.33, *P* = 0.02).

## Discussion

This is the first study assessing longitudinally the relationship between cerebral perfusion- or oxygenation parameters and short-term neurological outcome in infants with prenatally diagnosed CHD. The study demonstrates that prenatal Doppler flow patterns indicative of preferential brain perfusion are associated with poorer short-term neurological outcome in infants with CHD. Furthermore, it suggests that abnormal short-term neurological outcomes are likely the result of a cumulative effect of hypoxic-ischemic events during the prenatal period and early postnatal life. Infants with abnormal perfusion or oxygenation both prenatally as well as postnatally had a nine-fold increased risk of abnormal short-term neurological outcome in comparison with infants with no abnormal cerebral findings or abnormal cerebral findings only prenatally or postnatally.

Assessment of GMs is a widely accepted non-invasive method to determine the integrity of the central nervous system of the newborn [39]. At present, GMs are considered the best available method to assess short-term neurological outcome with a high sensitivity and specificity. Little is known about the quality of GMs in a CHD population. More is known on GMs in preterm born infants and, to a lesser extent, in term born infants [36,40–43]. The predictive value for neurological outcome of poor repertoire during the writhing period (around term age) is low [44]. However, absence of fidgety movements at an age of 3 months is strongly associated with adverse neurological outcome at school age [36,41]. Furthermore, abnormal MOS at an age of 3 months is also associated with motor impairments and minor neurological dysfunction at school age[40,42,43].

In our study, abnormal prenatal Doppler flow patterns indicative of preferential brain perfusion were associated with poorer short-term neurological outcome. Previous studies have reported contradictory results concerning the association between prenatal Doppler flow patterns and neurodevelopmental outcome in infants with CHD. Two studies found a negative association between MCA-PI and psychomotor developmental index evaluated by the Bayley scale of infant development II (BSID II) [7,45]. One study did not find any association between MCA-PI and neurodevelopmental outcome and another study found a positive correlation between MCA-PI and cognition measured by Bayley scale of infants and toddler development III (Bayley III) [5,46]. These contradictory findings may be explained by differences in study design (first vs. last Doppler measurement during pregnancy) and differences in cardiac lesions included in the study (different types of CHD with different circulatory and pathophysiological effects).

Our results indicate that Doppler patterns indicative for compensatory “brain sparing” mechanisms during fetal life actually suggest that oxygen delivery to the brain is insufficient to meet metabolic demands in fetuses with CHD. This may lead to impaired brain maturation, a finding that has been commonly reported in fetuses and neonates with CHD [10–15]. The preterm brain is likely to be more susceptible to hypoxic-ischemic events, explaining why fetuses with abnormal Doppler flow patterns are more prone to have abnormal short-term neurological outcomes [47].

While r_c_SO_2_ during the first 3 days was consistently lower and FTOE higher in infants with abnormal MOS at 3 months of age, we were unable to demonstrate a statistically significant association between preoperative cerebral oxygenation and developmental outcome. It might be that there is no association between preoperative oxygenation and short-term neurological outcome in infants with prenatally diagnosed CHD. We assessed neurological outcome at 3 months of age using the best available method at present, with a high sensitivity and specificity. We speculate that the brain may, at least transiently, be able to recover from hypoxic-ischemic events suffered immediately after birth. Brain development continues throughout childhood and involves not only the onset of new pathways and connections, but also elimination of others [48]. Due to the high plasticity of the young brain, previous abnormalities might disappear, at least transiently. It might also be that preoperative r_c_SO_2_ is associated with developmental outcome, but that our sample size was too small to reach statistical significance. Previous studies on the association between preoperative r_c_SO_2_ and neurodevelopmental outcome in infants with CHD, however, support our first explanation, as they were also unable to demonstrate a clear association between preoperative r_c_SO_2_ and neurodevelopmental outcome [49–50].

Cerebral oxygen saturation and FTOE during and after cardiac surgery were not associated with short-term neurological outcome. This is in line with previous literature[48–51]. Although some of these studies found an association between immediate preoperative, intraoperative or postoperative r_c_SO_2_ and various domains of neurodevelopmental outcome, r_c_SO_2_ was never a strong predictor of neurodevelopmental outcome [49–52]. Advanced surgical techniques and postoperative ICU care might prevent additional brain injury.

As we found that infants with abnormal perfusion or oxygenation both prenatally and postnatally had a nine-fold increased risk of having abnormal short-term neurological outcome, we suggest that there is a cumulative effect of hypoxic-ischemic events in infants with prenatally diagnosed CHD. This multiple-hit theory has been previously described in very preterm born infants [53]. Abnormal Doppler flow patterns before birth could have increased vulnerability of the brain to hypoxic-ischemic events after birth. Impaired brain maturation might be an important contributor to this vulnerability [11–16].

This study has several strengths and limitations. The longitudinal design from prenatal diagnosis until the age of 3 months is unique and important since infants with CH D are at risk of developing brain injury at various moments during early life. The observational design, however, implied that some of the values were missing, which might have induced selection bias. Other limitations were the relatively small sample size and the heterogeneity of the study population. The included types of CHD might have different effects on cerebral oxygenation and perfusion and they are associated with a different postnatal course (e.g. some infants required multiple surgeries during the first 3 months, while others did not require surgery). The infants with a more intense treatment course might have had less opportunity to develop certain age-appropriate skills. This could have influenced GMs at an age of 3 months. Nonetheless, our study population is a representative sample of an ICU and all included lesions have been associated with neurodevelopmental impairments later in life [1]. Furthermore, we did not have neuro-imaging data which could have confirmed brain injury or impaired brain maturation to support our vulnerability hypothesis in infants with abnormal GMs. In addition, we assessed short-term neurological outcome at only 3 months of age using GMs and MOS. The prognostic value of GMs and MOS is excellent for cerebral palsy and very good for mild motor abnormalities in preterm and healthy term infants. Less is known about the prognostic value of GMs and MOS in infants with CHD. Furthermore the prognostic value for cognitive deficits later on is less strong, which are a prominent finding in this population. Future studies should address these limitations and include enough patients to stratify for cardiac lesion. It is also crucial to extend neurodevelopmental testing to an older age in order to determine whether the associations we found persist later in infancy and childhood. Furthermore, future studies should address the cumulative effect of hypoxic-ischemic events, including multiple surgical procedures. This is also known as the multiple hit theory. We hope, with this study, to set the tone for these future larger (multicenter) longitudinal studies.

In conclusion, based on our findings we hypothesize that the prenatal period may play an important role in developmental outcome in infants with CHD that were admitted to the ICU immediately after birth. Additional research is needed to clarify the association between cerebral oxygenation during the first days after birth and neurological outcome in infants with CHD.

## Supporting information

S1 Table Type of CHD. MA, mitral atresia; AA, aortic atresia.
(DOCX)

S2 Table Fetal Doppler flow patterns, postnatal r_c_SO_2_ and FTOE according to GMs assessment at the age of three months. Data are presented as median (range). MOS, motor optimality score; MCA-PI, pulsatility index of the middle cerebral artery; UA-PI, pulsatility index of the umbilical artery; CPR, cerebroplacental ratio; r_c_SO_2_, cerebral oxygen saturation; FTOE, cerebral fractional tissue oxygen extraction. * indicates P-value <0.05.
(DOCX)

S1 Data (SAV)
