# Supplementary material for: Onset of brain injury in infants with prenatally diagnosed congenital heart disease
Source: PLoS One. 2020 Mar 25;15(3):e0230414. doi: 10.1371/journal.pone.0230414 (PMC7094875; doi:10.1371/journal.pone.0230414)
Supplement: S1 Table — MA, mitral atresia; AA, aortic atresia. (DOCX) [file pone.0230414.s001.docx]

**Supplemental table 1** **Type of CHD**

| **NR** | **Diagnosis** |
| --- | --- |
| 1 | Pulmonary atresia with intact ventricular septum and ventricular-coronary fistulas |
| 2 | Transposition of the great arteries with intact ventricular septum |
| 3 | Critical subvalvular pulmonary stenosis |
| 4 | Monoventricular heart, transposition of the great arteries, complete atrioventricular septal defect, subvalvular pulmonary stenosis |
| 5 | Truncus arteriosus type I |
| 6 | Coarctation of the aorta and ventricular septal defect |
| 7 | Transposition of the great arteries with intact ventricular septum |
| 8 | Transposition of the great arteries with ventricular septal defect |
| 9 | Hypoplastic left ventricle, critical aortic valve stenosis, coarctation of the aorta, hypoplastic aortic arch |
| 10 | Tetralogy of Fallot |
| 11 | Transposition of the great arteries with intact ventricular septum |
| 12 | Tetralogy of Fallot |
| 13 | Left atrial isomerism, complete atrioventricular septal defect |
| 14 | Double outlet right ventricle (Taussig-Bing), subvalvular aortic stenosis, hypoplastic aortic arch |
| 15 | Transposition of the great arteries with intact ventricular septum |
| 16 | Complete atrioventricular septal defect |
| 17 | Coarctation of the aorta, hypoplastic aortic arch, ventricular septal defect |
| 18 | Hypoplastic left heart syndrome (MA/AA) |
| 19 | Coarctation of the aorta, ventricular septal defect |
| 20 | Tricuspid atresia with right ventricle hypoplasia, transposition of the great ventricles, ventricular septal defect, coarctation of the aorta, hypoplastic aortic arch |
| 21 | Transposition of the great arteries with intact ventricular septum |
| 22 | Pulmonary atresia, Ebstein anomaly |
| 23 | Transposition of the great arteries with intact ventricular septum |
| 24 | Tetralogy of Fallot |
| 25 | Right atrial isomerism, atrioventricular septal defect, pulmonary atresia, transposition of the great arteries |
| 26 | Right atrial isomerism, atrioventricular septal defect, pulmonary atresia |
| 27 | Transposition of the great arteries with intact ventricular septum |
| 28 | Double inlet left ventricle, transposition of the great arteries, interruption of the aortic arch type A |
| 29 | Transposition of the great arteries with ventricular septal defect |
| 30 | Unbalanced right-dominant atrioventricular septal defect. |
| 31 | Double outlet right ventricle, transposition of the great arteries, hypoplastic aortic arch |
| 32 | Common arterial trunk type I |
| 33 | Tetralogy of Fallot |
| 34 | Common arterial trunk type I |
| 35 | Dysplastic tricuspid valve, atrial septal defect, ventricular septal defect, small left ventricle |
| 36 | Coarctation of the aorta |

*MA, mitral atresia; AA, aortic atresia;*
