# Supplementary material for: Onset of brain injury in infants with prenatally diagnosed congenital heart disease
Source: PLoS One. 2020 Mar 25;15(3):e0230414. doi: 10.1371/journal.pone.0230414 (PMC7094875; doi:10.1371/journal.pone.0230414)
Supplement: S2 Table — Data are presented as median (range). MOS, motor optimality score; MCA-PI, pulsatility index of the middle cerebral artery; UA-PI, pulsatility index of the umbilical artery; CPR, cerebroplacental ratio; rcSO2, cerebral oxygen saturation; FTOE, cerebral fractional tissue oxygen extraction. * indicates P-value <0.05. (DOCX) [file pone.0230414.s002.docx]

**Supplemental table 2** **Fetal Doppler flow patterns, postnatal r­_c_SO­_2_ and FTOE according to GMs assessment at the age of three months**

| **Variable** | **Normal MOS** | **Abnormal MOS** |
| --- | --- | --- |
| MCA-PI | 0.48 (-1.44 to 1.99) | -1.65 (-3.94 to 1.57)* |
| UA-PI | 0.14 (-1.48 to 1.03) | 1.34 (-1.74 to 1.98)* |
| CPR | -0.12 (-1.44 to 2.88) | -1.55 (-3.84 to1.20)* |
| R_c_SO_2_ day 1 | 68 (34-89) | 60 (34-81) |
| R_c_SO_2_ day 2 | 72 (42-89) | 67 (48-75) |
| R_c_SO_2_ day 3 | 72 (52-91) | 68 (52-85) |
| R_c_SO_2_ during surgery | 55 (52-62) | 53 (36-56) |
| R_c_SO_2_ day after surgery | 59 (54-79) | 55 (52-65) |
| FTOE day 1 | 0.27 (0.05-0.59) | 0.36 (0.14-0.61) |
| FTOE day 2 | 0.24 (0.05-0.53) | 0.29 (0.19-0.48)* |
| FTOE day 3 | 0.21 (0.02-0.40) | 0.29 (0.14-0.47) |
| FTOE during surgery | 0.41 (0.29-0.44) | 0.45 (0.38-0.61) |
| FTOE after surgery | 0.38 (0.30-0.42) | 0.33 (0.27-0.39) |

*Data are presented as median (range). MOS, motor optimality score; MCA-PI, pulsatility index of the middle cerebral artery; UA-PI, pulsatility index of the umbilical artery; CPR, cerebroplacental ratio; r_c_SO_2_,, cerebral oxygen saturation; FTOE, cerebral fractional tissue oxygen extraction. * indicates P-value <0.05.*
